# Post-traumatic Stress Disorder and Risk Factors in Patients With Acute Myocardial Infarction After Emergency Percutaneous Coronary Intervention: A Longitudinal Study

**DOI:** 10.3389/fpsyg.2021.694974

**Published:** 2021-12-14

**Authors:** Xiaocui Cao, Jiaqi Wu, Yuqin Gu, Xuemei Liu, Yaping Deng, Chunhua Ma

**Affiliations:** ^1^Department of Cardiovascular Medicine, The Second Affiliated Hospital of Guangzhou Medical University, Guangzhou, China; ^2^School of Nursing, Guangzhou Medical University, Guangzhou, China

**Keywords:** acute myocardial infarction, anxiety, depression, percutaneous coronary intervention, post-traumatic stress disorder

## Abstract

This study aimed to investigate the status and risk factors of post-traumatic stress disorder (PTSD) in patients with acute myocardial infarction (AMI) after emergency percutaneous coronary intervention (PCI) in acute and convalescence phases. A longitudinal study design was used. Two questionnaire surveys were conducted in the acute stage of hospitalization, and 3 months after onset in patients. Logistic regression was used to analyze the risk factors for PTSD in AMI patients. The incidence of PTSD was 33.1 and 20.4% in acute and convalescent patients, respectively. The risk factors related to PTSD were door-to-balloon time (DTB) (≥92.6 min), left ventricular ejection fraction (LVEF) (<50%), smoking, anxiety, and depression. AMI patients after PCI had PTSD in the acute and convalescent stage. The findings indicate that tailored measures should be developed and carried out to prevent PTSD and improve the mental health of patients with AMI after undergoing PCI.

## Introduction

Acute myocardial infarction (AMI) is a common and serious heart disease with rapid onset, extremely high morbidity, and mortality (Reed et al., 2017). Approximately 50% of AMI patients have multivessel coronary artery disease (Saito and Kobayashi, 2019). The key to successful AMI treatment is to open the infarct-related artery as soon as possible, and emergency percutaneous coronary intervention (PCI) is the mainstay of treatment for AMI patients (Levine et al., 2016). Post-traumatic stress disorder (PTSD) is defined as a stress-related disorder with a subsequent autoimmune disease that might arise after exposure to a serious traumatic event or injury (Liang et al., 2020). Previous studies have shown that approximately 12% of AMI patients developed PTSD (Edmondson et al., 2012), and 66.7% of patients had PTSD symptoms 2 years after AMI (Castilla and Vázquez, 2011). The abruptness of the event, the risk of death, and the patient’s intense sense of loss of control and helplessness during the event as well as the intrusive experience of the treatments, such as PCI, could lead to the development of PTSD (Ledermann et al., 2020). In addition, the occurrence of PTSD after emergency PCI is associated with poor therapeutic efficacy, such as repeated rehospitalization, multiple complications, and increased mortality (Guler et al., 2009; Edmondson et al., 2012; Sumner et al., 2015; Burg and Soufer, 2016). Therefore, approximately 18–23% of patients with AMI, who perceived the event as life-threatening and distressing, showed clinically relevant acute stress symptoms (Ledermann et al., 2020). AMI is a stressful traumatic event, and AMI patients usually have a severe psychologically traumatic experience that endangers their lives. Recently, an increasing number of studies have explored AMI-related PTSD (Edmondson et al., 2011; Perkins-Porras et al., 2015; Singh et al., 2017; Birk et al., 2019). Previous study (Meli et al., 2019) showed that invasive treatment was a risk factor for PTSD in AMI patients, and AMI patients that received invasive surgery were more prone to develop PTSD symptoms than those that received conservative treatment. PTSD after AMI could cause prolonged psychological pain and increase the risk of major adverse cardiac events, which results in impaired quality of life (Känel et al., 2011; Edmondson and von Känel, 2017).

Some studies found that the occurrence of PTSD in AMI patients might be related to the patient’s social demographic factors (e.g., age, gender, and race), disease factors, socioeconomic factors, psychological factors (e.g., anxiety and depression), and personality (e.g., introversion and impulsive personality traits) (Wikman et al., 2008; Hari et al., 2010; Roberge et al., 2010; Dinenberg et al., 2014; Oflaz et al., 2014; Stevanović et al., 2016). Previous studies found that depression was strongly associated with the development of PTSD and comorbidity and that depression (hospitalization or before onset) was predictive of PTSD (Whitehead et al., 2006; Roberge et al., 2010). Konrad et al. (2017) reported that several environmental factors in emergency departments influenced a patient’s perception of threat to life, and increased risk for subsequent PTSD in AMI patients. Many patients showed a strong fear of dying after the onset of acute symptoms, and emotional distress is common in approximately one-third of patients that reported mild-to-moderate depressive symptoms post-hospitalization for AMI (Wikman et al., 2008).

Currently, the number of AMI patients after discharge that have PSTD symptoms is difficult to assess, and the diversity of symptoms means that it is difficult to determine the progression of PSTD and evaluate the effectiveness of treatment. Therefore, this study adopted a longitudinal study design to investigate PTSD and the risk factors in AMI patients following emergency PCI during the in-hospital acute stage and at-home convalescent stage. The results could mean that is it possible to identify patients at risk and help to improve postoperative nursing.

## Materials and Methods

### Study Design and Procedures

A longitudinal design was used in this study. AMI patients that were admitted to the University Hospital, Guangzhou, Southern China, between September 1, 2019, and March 31, 2020, and were successfully implanted with coronary stents by emergency PCI were enrolled in this study.

Patients that had all the following were included in this study: (1) AMI and emergency PCI; (2) aged ≥ 18 years; (3) no other major traumatic events in the previous 6 months, such as car accidents and family changes; (4) willingness to participate in the survey; and (5) clear consciousness, no cognitive impairment, capacity to communicate using language or text. The following patients were excluded from this study: (1) patients that needed to be transferred to ICU for treatment due to serious illness after PCI; (2) patients that had severe hepatic and renal insufficiency and malignant tumors; (3) patients that had a previous mental illness, such as dementia, anxiety, depression, or a family history of mental illness; (4) patients that had a respiratory infection, urinary tract infection, and other serious infectious diseases, and (5) patients that received coronary artery bypass graft surgery.

The sample size of this study was estimated based on the previous study (Xiao, 2008). The PTSD scale consisted of 17 items, each item for five cases at least, considering 15% non-response, a minimum of sample size was 98. 113 AMI patients completed two times survey, which achieved the requirements of sample size.

### Instruments

#### Demographic and Baseline Clinical Data

Demographic and baseline clinical data for patients included gender, age, level of education, marital status, smoking history, body mass index (BMI), baseline clinical characteristics (e.g., history of hypertension, diabetes, and hyperlipidemia), chest pain center time index and door-to-balloon (DTB), infarction area, number of coronary artery stenoses, and left ventricular ejection fraction (LVEF).

#### PTSD Checklist-Civilian Version

The scale (Känel et al., 2011) to evaluate PTSD was developed by the PTSD research center in the United States, which includes three characteristic syndromes: (1) re-experience (5 items); (2) avoidance (7 items); and (3) hypervigilance (5 items), with 17 items in total (Li et al., 2010). Each item was used with a five-point Likert scale, with a total score from 17 to 85. The higher the score, the more probable the patient was to develop PTSD. Cronbach’s regression coefficient for the scale was 0.87. According to a previous study, a total score ≥ 44 suggested the presence of the symptoms of PTSD (Li et al., 2010).

#### Hospital Anxiety and Depression Scale

The hospital anxiety and depression scale (HADS) was used to assess the mental state of patients. The HADS scale has good predictive accuracy for anxiety and depression patients and can be used for patients without the participation of psychologists or psychiatrists (Reed et al., 2017). The HADS consists of two subscales (1) anxiety (A); and (2) depression (D), each has seven items, and each item is divided into four grades from 0 to 3. A score from 0 to 7 indicated no depression or anxiety, from 8 to 10 indicated depression or anxiety, from 11 to 14 indicated possible moderate depression or anxiety, and from 15 to 21 indicated severe depression or anxiety. Cronbach’s regression coefficient for the scale was 0.83.

### Procedure

Acute myocardial infarction patients that underwent emergency PCI were screened by two trained research assistants based on electronic medical records and clinical recommendations. Researchers conducted unified training for investigators before data collection. Patients that had a lower educational level, older age, or visual impairment had each item explained in detail to ensure that they understood the meaning of each item clearly.

The first survey was conducted between days 1 and 3 after patients were stabilized during the acute hospitalization stage after emergency PCI. Face-to-face PCL-C and HADS questionnaires were conducted and the participants’ medical record number and telephone number were recorded to track the patient’s condition. The second survey was conducted during at-home convalescence (3 months after the onset of the disease). PCL-C and HADS questionnaires were conducted again by telephone. Each survey lasted approximately 5–10 min, and the investigators only asked questions and explained the meaning of the questions; however, they did not help the patients to make the choices.

### Statistical Analyses

SPSS 20.0 statistical software was used for data analysis. Data processing included the elimination of questionnaires that had an information missing rate of > 10% or that had the same answers but with obvious problems. Continuous variables were described by mean and standard deviation, and classified variables were described by frequency and percentage. Continuous variables and categorical variables were compared using a *t*-test and a Chi-squared test, respectively. Associated risk factors for PTSD were analyzed using a logistic regression model. All hypothesis tests were bilateral tests. A *p*-value < 0.05 indicated that the difference was statistically significant.

## Results

### General Demographic Characteristics

After application of the inclusion and exclusion criteria, a total of 129 AMI patients were enrolled in this study. In this study, at stage T1, 129 questionnaires were issued, and 121 valid questionnaires were collected, eight questionnaires had missing information, which included three patients that had missing HADS data, three patients that had missing PCL-C data, and two patients that had experienced deteriorating conditions and had been transferred to ICU for treatment. Therefore, 121 patients presented valid questionnaires at this stage. At stage T2, 113 valid questionnaires were followed up by telephone, eight patients were lost during follow-up. Among eight lost participants, one patient died in the ICU because of illness deterioration, four patients did not answer the telephone, and three patients could not complete the questionnaire by telephone.

At stage T1, the 121 patients included 103 (85.1%) males and 18 (14.9%) females with an average age of 61.2 ± 10.7 years (age range: 36–85 years). Among these 121 patients, 115 patients (95.0%) had ST segment elevation myocardial infarction (STEMI) and six patients (5.0%) had non-ST segment elevation myocardial infarction (NSTEMI). At stage T2, the 113 patients included 95 (84.0%) males and 18 (26.0%) females with an average age of 63.7 ± 10.0 years (age range: 38–84 years). Among these 113 patients, 107 patients (94.7%) had STMEI and six patients (5.3%) had NSTEMI (Table 1).

**TABLE 1:** Demographic and baseline clinical data of patients during acute and convalescent stages.

Variables	AMI patients (*n* = 121)	PTSD during acute stage (*n* = 121)			PTSD during convalescent stage (*n* = 113)		
		No (*n* = 81)	Yes (*n* = 40)	*p* *	No (*n* = 90)	Yes (*n* = 23)	*p* *
Age	61.2 ± 10.7	64.3 ± 9.5	54.8 ± 10.3	<0.001	62.8 ± 10.2	52.9 ± 8.9	<0.001
**Gender (age)**				0.109			0.043
Male	103 (85.1)	66 (81.5)	37 (92.5)		72 (80.0)	23 (100)	
Female	18 (14.9)	15 (18.5)	3 (7.5)		18 (18.5)	0 (0)	
BMI (kg/m^2^)	24.1 ± 1.9	24.0 ± 2.0	24.3 ± 1.8	0.414	24.0 ± 1.9	24.3 ± 2.0	0.629
**Degree of education**				<0.001			0.002
Junior high school or below	73 (60.3)	60 (74.1)	13 (32.5)		60 (66.7)	7 (30.4)	
High school or technical secondary school	48 (39.7)	21 (25.9)	27 (67.5)		30 (33.3)	16 (69.6)	
**Marital status**				0.631			0.287
Married	100 (82.6)	66 (81.5)	34 (85.0)		71 (78.9)	21 (91.3)	
Divorced or widowed	21 (17.4)	15 (18.5)	6 (15.0)		19 (21.1)	2 (8.7)	
**Smoking**				<0.001			<0.001
No	65 (53.7)	53 (65.4)	12 (30.0)		57 (63.3)	4 (17.4)	
Yes	56 (46.3)	28 (34.6)	28 (70.0)		33 (36.7)	19 (82.6)	
**Disease diagnosis**				0.177			0.452
STEMI	115 (95)	79 (97.5)	36 (90.0)		84 (93.3)	23 (100)	
NSTEMI	6 (5)	2 (2.5)	4 (10.0)		6 (6.7)	0 (0)	
FMC (min)	545.2 ± 918.9	407.6 ± 677.7	823.8 ± 1,239.7	0.053	595.4 ± 994.3	379.2 ± 573.6	0.320
DTB (min)	92.6 ± 56.12	69.3 ± 23.4	139.6 ± 72.0	<0.001	89.2 ± 49.8	112.8 ± 79.2	0.185
**Number of coronary artery stenoses**				0.159			0.815
Single branch	41 (33.9)	24 (29.6)	17 (42.5)		29 (32.2)	8 (34.8)	
Multiple branches	80 (66.1)	57 (70.4)	23 (57.5)		61 (67.8)	15 (65.2)	
**Area of infarction**				0.202			0.969
Anterior wall	38 (31.4)	22 (27.2)	16 (40.0)		28 (31.1)	8 (34.8)	
Inferior wall	46 (38.0)	36 (44.4)	10 (25.0)		34 (37.8)	8 (34.8)	
Multiple sites	25 (20.7)	15 (18.5)	10 (25.0)		18 (20.0)	5 (21.7)	
Other sites	12 (9.9)	8 (9.9)	4 (10.0)		10 (11.1)	2 (8.7)	
**LVEF**				0.001			<0.001
<50%	34 (28.1)	15 (18.5)	19 (47.5)		17 (18.9)	15 (65.2)	
≥50%	87 (71.9)	66 (81.5)	21 (52.5)		73 (81.1)	8 (34.8)	
**Hypertension**				0.404			0.112
No	67 (55.4)	47 (58.0)	20 (50.0)		46 (51.1)	16 (69.6)	
Yes	54 (44.6)	34 (42.0)	20 (50.0)		44 (48.9)	7 (30.4)	
**Diabetes mellitus**				0.390			0.675
No	79 (63.2)	55 (67.9)	24 (60.0)		59 (65.6)	14 (60.9)	
Yes	42 (34.7)	26 (32.1)	16 (40.0)		31 (34.4)	9 (39.1)	
**Hyperlipidemia**				0.653			0.076
No	76 (62.8)	52 (64.2)	24 (60.0)		61 (67.8)	11 (47.8)	
Yes	45 (37.2)	29 (35.8)	16 (40.0)		29 (32.2)	12 (52.2)	

*p < 0.001, paired t-tests.

### Acute and Convalescent Stages of Post-traumatic Stress Disorder

Out of 121 patients in the acute stage, 40 had PTSD, with an incidence rate of 33.1% and a PTSD average score of 35.71 ± 7.52. Out of 113 patients during the convalescent stage, 23 had PTSD, with an incidence rate of 20.4% and a PTSD average score of 28.95 ± 8.4.

### Analysis of Anxiety and Depression in Acute and Convalescent Stages

**FIGURE 1:**
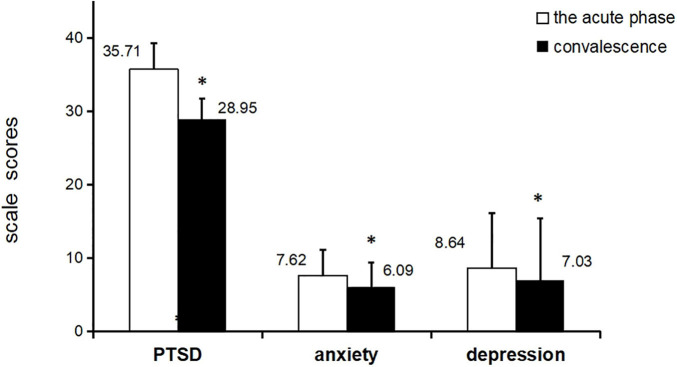
The average scores of PTSD, anxiety, and depression in acute and convalescent phase *p<0.001, paired t-test, PTSD=post traumatic stress disorder.

During the acute stage, 121 patients had average scores of anxiety and depression of 7.62 ± 3.56 and 8.64 ± 3.54, respectively, with rates of anxiety and depression of 46.3 and 46.3%, respectively. During the convalescent stage, 113 patients had average scores of anxiety and depression of 6.09 ± 2.77 and 7.03 ± 3.33, respectively, with rates of anxiety and depression of 41.6 and 45.1%, respectively (Figure 1).

### Risk Factors for Post-traumatic Stress Disorder During Acute and Convalescent Stages

As listed in Table 2, during the acute stage, univariate analysis of PTSD revealed that the risk factors for PTSD included young age [OR = 0.18, 95% CI: 0.08–0.41, *p* < 0.001], higher education level [OR = 5.93, 95% CI: 2.59–13.57, *p* < 0.001], smoking [OR = 4.42, 95% CI: 1.95–10.00, *p* < 0.001], DTB ≥ 92.6 min [OR = 11.94, 95% CI: 4.85–29.43, *p* < 0.001], and LVEF < 50% [OR = 0.25, 95% CI: 0.11–0.58, *p* = 0.001]. Binary logistic analysis suggested that DTB ≥ 92.6 min [OR = 34.83, 95% CI: 8.61–141.00, *p* < 0.001] and LVEF <50% [OR = 0.08, 95% CI: 0.02–0.30), *p* < 0.001] were independent risk factors for PTSD in AMI patients.

**TABLE 2:** Determination of risk factors for PTSD during acute stages.

Variables		Univariate analysis		Multifactor analysis	
		OR (95% CI)	*P* *	OR (95% CI)	*P* *
Age					
	< 61.2	1.00 (Ref.)		1.00 (Ref.)	
	≥ 61.2	0.18 (0.08–0.41)	< 0.001	0.23 (0.05–1.15)	0.073
Degree of education					
	Junior high school and below	1.00 (Ref.)		1.00 (Ref.)	
	Senior high school and above	5.93 (2.59–13.57)	< 0.001	3.06 (0.62–15.01)	0.169
Smoking					
	No	1.00 (Ref.)		1.00 (Ref.)	
	Yes	4.42 (1.95–10.00)	< 0.001	2.77 (0.75–10.22)	0.125
DTB (min)					
	< 92.6	1.00 (Ref.)		1.00 (Ref.)	
	≥ 92.6	11.94 (4.85–29.43)	< 0.001	34.83 (8.61–141.00)	< 0.001
LVEF					
	< 50%	1.00 (Ref.)		1.00 (Ref.)	
	≥ 50%	0.25 (0.11–0.58)	0.001	0.08 (0.02–0.30)	< 0.001

*p < 0.001, paired *t*-tests.

*DTB, door-to-balloon; LVEF, left ventricular ejection fraction.*

During the convalescent stage, univariate analysis revealed that the risk factors for PTSD included young age [OR = 0.20, 95% CI: 0.07–0.60, *p* = 0.004], higher education level [OR = 4.57,95% CI: 1.7–12.31, *p* = 0.003], smoking [OR = 8.21, 95% CI: 2.57–26.18, *p* < 0.001], and LVEF < 50% [OR = 0.12, 95% CI: 0.05–0.34, *p* < 0.001]. Multiple factor logistic regression analysis suggested that smoking [OR = 5.12, 95% CI: 1.30–20.16, *p* = 0.019] and LVEF<50% [OR = 0.08, 95% CI: 0.02–0.28), *p* < 0.001] were the independent risk factors for PTSD in AMI patients (Tables 2, 3).

**TABLE 3:** Determination of risk factors for PTSD during convalescent stages.

Variables		Univariate analysis		Multifactor analysis	
		OR (95% CI)	*P* *	OR (95% CI)	*P* *
Age					
	< 61.2	1.00 (Ref.)		1.00 (Ref.)	
	≥ 61.2	0.20 (0.07–0.60)	0.004	0.40 (0.07–2.23)	0.297
Degree of education					
	Junior high school and below	1.00 (Ref.)		1.00 (Ref.)	
	Senior high school and above	4.57 (1.70–12.31)	0.003	2.35 (0.46–12.07)	0.307
Smoking					
	No	1.00 (Ref.)		1.00 (Ref.)	
	Yes	8.21 (2.57–26.18)	< 0.001	5.12 (1.30–20.16)	0.019
LVEF					
	< 50%	1.00 (Ref.)		1.00 (Ref.)	
	≥ 50%	0.12 (0.05–0.34)	< 0.001	0.08 (0.02–0.28)	< 0.001

*p < 0.001, paired t-tests.

*DTB, door-to-balloon; LVEF, left ventricular ejection fraction.*

### Relationship Between Anxiety, Depression, and Post-traumatic Stress Disorder

**FIGURE 2:**
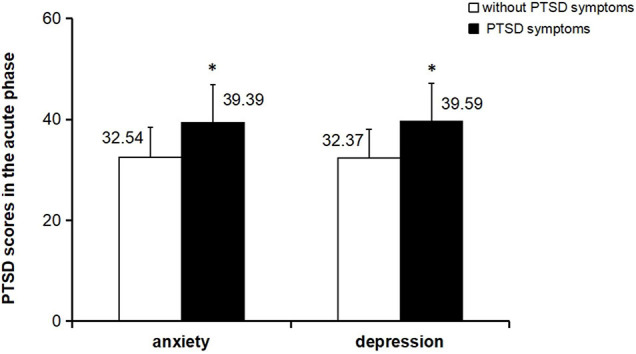
Comparison of PTSD scores between anxiety and depression subgroups in the acute phase *p<0.001 paired t-test.

The probability of PTSD symptoms in patients with anxiety and depression symptoms were compared and patients with anxiety and depression symptoms were more probable to have PTSD symptoms (Figure 2 and Table 4).

**TABLE 4:** Comparison of the positive rates of PTSD between anxiety and depression subgroups during the acute stage.

Index		PTSD during acute stage (*n* = 121)		*P* *
		No (*n* = 81)	Yes (*n* = 40)	
Anxiety				< 0.001
	Yes (*n* = 65)	59 (90.8)	6 (9.2)	
	No (*n* = 56)	22 (39.3)	34 (60.7)	
Depression				< 0.001
	No (*n* = 65)	60 (92.3)	5 (7.7)	
	Yes (*n* = 56)	21 (37.5)	35 (62.5)	

*Chi-square tests.

## Discussion

In this study, the incidence of PTSD in AMI patients following PCI was 33.1% during the acute stage and 20.4% during the at-home recovery stage 3 months after discharge. Compared with the recovery period, the incidence of PTSD in the acute phase was higher in AMI patients after emergency PCI. A systematic review and meta-analysis showed that the incidence of PTSD after acute coronary syndromes was approximately 12%, with a prevalence rate from 0 to 32% in each study (Edmondson et al., 2012). The differences between these observations and data available in the literature could be attributed to the differences in the methods used to evaluate PTSD and the sample size. In this study, the AMI patients that had emergency PCI during the acute and convalescent stages had a higher incidence of PTSD, which was probably affected by the COVID-19 pandemic (i.e., increased isolation) during the assessment period. Because of COVID-19, AMI patients following PCI worried that they, their family members, and friends might be infected. Therefore, they experienced more psychological pressure and were more probable to develop PTSD (Edmondson et al., 2012; Vindegaard and Benros, 2020). Although the proportion of patients with PTSD decreased slightly during the recovery period, patients were not fully disengaged from the traumatic event. Therefore, AMI is a persistent potential threat, and patients might experience AMI-related symptoms after onset, which might last for many years (Wikman et al., 2008). These observations suggest that medical staff should pay more attention to the early mental and psychological health of AMI patients after emergency PCI and if they have PTSD symptoms. If required, psychological and other medical treatments should be administered to patients that have obvious PTSD symptoms to promote early recovery.

Multivariate analysis of PTSD during the acute stage showed that the longer the DTB time, the increased risk of PTSD. In 2013, The guidelines emphasized that DTB duration for STEMI patients that received PCI should be < 90 min (O’gara et al., 2013). The guidelines for myocardial revascularization published in 2014 suggested that the appropriate DTB time was < 60 min (Kolh and Windecker, 2014). Therefore, DTB time is the most critical time index during the treatment procedure (O’gara et al., 2013; Kolh and Windecker, 2014), and reopening the blood supply in the shortest time to achieve myocardial level reperfusion could save more functional myocardium. The severity of AMI is a risk factor for PTSD (Xia Liu et al., 2019). A shorter DTB might remove the patient from danger more quickly; therefore, reducing the psychological burden as well as the probability of PTSD, anxiety, and depression. Therefore, rapid revascularization and reduction of DTB time during the treatment of AMI patients is critical to reduce the incidence of PTSD.

The results of this study indicated that LVEF < 50% was a risk factor for PTSD. LVEF is an important indicator for the evaluation of cardiac function (Jia et al., 2011). The degree of myocardial ischemia in STEMI patients depends on the time of vascular opening and delayed vascular opening results in massive cardiac cell death, myocardial infarction, left ventricular remodeling, and a continuous decrease in LVEF. A larger scope for myocardial necrosis has an increased negative impact on cardiac function and blood circulation dynamics, and therefore, generates more somatic symptoms, and places increased psychological stress on AMI patients. Newman et al. (2011) surveyed 241 patients with acute coronary syndromes and reported that those with PTSD symptoms (18%) were higher than those without PTSD symptoms (82%) (LVEF: 53.0 vs. 46.1%). In addition, (Sawatari et al., 2016) found that LVEF level was a useful to predict of PTSD severity. Therefore, early active treatment should be carried out to improve the cardiac function of severe AMI patients to promote the early recovery, reduce psychological stress, and prevent, or reduce PTSD, or both.

In this study, patients that were smokers were more probable to develop PTSD, which agreed with previous research (Calhoun et al., 2008). AMI patients were admitted to the Coronary Care Unit after emergency PCI and were not allowed to smoke, and eventually had to stop smoking. Because nicotine enhances cognition and attention, giving up smoking might increase the cognitive and attention deficit associated with PTSD. Compared with smokers without PTSD, smokers with PTSD had a higher rate of relapse aspiration and withdrawal syndrome during cessation, and their tolerance for pain associated with the withdrawal threshold was low, and the trauma of withdrawal was more sensitive (Dedert et al., 2012; Ashare et al., 2014; Tidey and Miller, 2015). Therefore, smoking cessation guidance for AMI patients should be strictly implemented during the acute hospitalization stage, and the possibility of PTSD should be assessed during the convalescent stage. In addition, during the convalescent stage, more support and intervention should be administered to AMI patients to help them stop smoking.

The development of PTSD in AMI patients was closely related to anxiety and depression during the acute and convalescent stages. AMI patients had increased anxiety and depression with different degrees before and after PCI surgery, probably because they were excessively worried about the risks of PCI itself and the potential complications and other uncertain factors (Langvik and Hjemdal, 2015). Previous studies concluded that depression and PTSD were reciprocal risk factors (Whitehead et al., 2006; Roberge et al., 2010). Therefore, when AMI patients display any signs of anxiety and depression after emergency PCI, nursing staff should closely monitor and evaluate if the patients develop PTSD during the acute and convalescent stages. When symptoms of anxiety, depression, and PTSD coexist, nursing staff should assess if symptoms of serious complications occur and offer early psychological intervention to prevent PTSD and to improve the prognosis of AMI patients.

Although age and education was not significant risk factors of PTSD in the multivariable model, young age or higher education level was associated with PTSD risk during the acute stage and the convalescent stage in the univariate analysis. Consistent with the previous research, patients with cancer who were younger reported to be a greater risk of PTSD (Abbey et al., 2015). Lin et al. (2017) showed the highly educated participants were likely to have PTSD. Therefore, AMI patients with young age or high education levels should be provided with health care to prevent PTSD after emergency percutaneous coronary intervention.

This study has some limitations. First, this study was a single-center study with a small sample size, therefore, caution should be employed when generalizing from the findings, and further research with a larger sample is needed to validate them. Second, the convalescent stage analyzed in this study was 3 months after discharge, which was relatively short. Therefore, future studies should have a follow-up for a longer recovery period to evaluate the occurrence of PTSD in AMI patients following PCI more accurately. In addition, the measurement methods used in this study were questionnaires that were completed by the patients or obtained by phone. Therefore, the possibility that the patients were subjective when answering questions could not be ruled out, which might not reflect their real psychological state.

## Conclusion

In this study, a higher incidence of PTSD was reported in AMI patients during the acute and convalescent stages after emergency PCI. The occurrence of PTSD was closely related to a long DTB time, LVEF < 50%, and smoking, and the AMI patients with PTSD had a higher comorbidity rate with anxiety and depression than those without PTSD. Therefore, attention should be paid to the mental health problems of AMI patients during the acute and convalescent stages after emergency PCI. During the acute stage, emergency care should be strengthened for AMI patients with early emergency PCI, DTB time should be shortened to reduce the incidence of PTSD, and smoking, poor heart function, and anxiety and depression symptoms should be closely monitored. During the convalescent stage, follow-up should be improved to closely monitor if AMI patients have PTSD symptoms after discharge, and suitable interventions should be conducted to reduce the adverse health outcomes associated with PTSD.

Future research should focus on the development of tools to screen and assess PTSD in AMI patients to determine the incidence and severity of PTSD more accurately. The follow-up time should be extended to assess PTSD at different time points after AMI to determine whether patients have PTSD after 6 months, 1 year, or a longer period after discharge from hospital. In addition, qualitative research should be designed to explore the causes of PTSD from the patients perspective to adopt more personalized interventions to prevent or reduce the occurrence of PTSD, reduce the adverse outcomes caused by PTSD, and improve the quality of life of patients.

## Data Availability Statement

The original contributions presented in the study are included in the article/supplementary material, further inquiries can be directed to the corresponding author/s.

## Ethics Statement

The studies involving human participants were reviewed and approved by the Ethics Committee of the Second Affiliated Hospital of Guangzhou Medical University. The patients/participants provided their written informed consent to participate in this study. Written informed consent was obtained from the individual(s) for the publication of any potentially identifiable images or data included in this article.

## Author Contributions

XC, JW, YG, XL, YD, and CM: conceptualization. XC and JW: methodology and writing—original draft preparation. YD and XL: formal analysis and investigation. JW and CM: writing—review and editing. XC and CM: funding acquisition and supervision. YG and XL: resources. XC and JW equally contributed to the writing of this article. All authors contributed to the article and approved the submitted version.

## Conflict of Interest

The authors declare that the research was conducted in the absence of any commercial or financial relationships that could be construed as a potential conflict of interest.

## Publisher’s Note

All claims expressed in this article are solely those of the authors and do not necessarily represent those of their affiliated organizations, or those of the publisher, the editors and the reviewers. Any product that may be evaluated in this article, or claim that may be made by its manufacturer, is not guaranteed or endorsed by the publisher.
